# A Novel Mutation of *SMAD3* Identified in a Chinese Family with Aneurysms-Osteoarthritis Syndrome

**DOI:** 10.1155/2015/968135

**Published:** 2015-06-29

**Authors:** Wenwen Zhang, Min Zhou, Cheng Liu, Chen Liu, Tong Qiao, Dian Huang, Feng Ran, Wei Wang, Changjian Liu, Zhao Liu

**Affiliations:** Department of Vascular Surgery, Nanjing Drum Tower Hospital, The Affiliated Hospital of Nanjing University Medical School, 321 Zhongshan Road, Nanjing, Jiangsu 210008, China

## Abstract

Aneurysms-osteoarthritis syndrome (AOS) is a recently delineated autosomal dominant disorder characterized by aneurysms, dissections, and tortuosity throughout the arterial tree in association with early onset osteoarthritis, mild craniofacial features, and skeletal and cutaneous anomalies. Previous studies have demonstrated that mutations in *SMAD3*, a key regulator of TGF-*β* signal transduction, contribute to AOS. Here, we investigated a family of three generations affected by AOS. A novel *SMAD3* mutation, c.266G>A (p.C89Y), was identified and cosegregated with the affected individuals in this family. Our finding expands the mutation spectrum of *SMAD3* gene and further strengthens the connection between the presence of aneurysms-osteoarthritis phenotype and *SMAD3* mutations, which facilitates the understanding of the genotype-phenotype correlation of AOS.

## 1. Introduction

Aneurysms-osteoarthritis syndrome (AOS) is an autosomal dominant disorder caused by mutation in the* SMAD3* gene encoding protein that is essential for TGF-*β* signal transduction [1, 2]. It is characterized by the presence of aneurysms, dissections, and tortuosity throughout the arterial tree in association with mild craniofacial, skeletal and cutaneous anomalies, and early onset osteoarthritis. AOS is a recently recognized disease and genotype-phenotype correlation remains yet to be determined. Patients may present with a wide spectrum of phenotypes, ranging from very mild (isolated bifid uvula) to severe (multiple aneurysms and dissections) [3]. Despite different genes involved, clinical features of AOS significantly overlap with other multisystem disorders such as Marfan syndrome (MFS), Loeys-Dietz syndrome (LDS), or vascular Ehlers-Danlos syndrome (vEDS) [2]. However, patients with AOS often suffer from early onset osteoarthritis, a distinguished manifestation that may direct molecular analysis [2, 4]. Moreover,* SMAD3* mutation carriers are more susceptible to early sudden death due to aortic dissection and/or rupture, which often occurs in a mildly dilated aorta [5].

Located on chromosome 15q22,* SMAD3* spans nine exons and encodes a 425 amino acid protein comprised of two major domains, the MH1 (MAD homology 1) domain and the MH2 (MAD homology 2) domain. To date, more than 20 pathogenic* SMAD3* sequence variants have been identified, including missense, nonsense, frameshift, and splice-site mutations [1–4, 6–12]. Most disease-causing mutations are clustered within exon 6 in the MH2 domain. Haploinsufficiency of SMAD3 is thought to play a critical role in the development of AOS [2]. However, augmented TGF-*β* signaling is observed in certain mutation carriers, which may be attributed to compensatory increase caused by functional haploinsufficiency of SMAD3 [2].

So far, AOS mutations have been only reported in Caucasian families. In the present study, we described the clinical findings from the study of a small Chinese family affected with AOS; the novel SMAD3 p.Cys89Tyr mutation was identified in the affected individuals.

## 2. Patients and Methods

### 2.1. Patients

**Figure 1:**
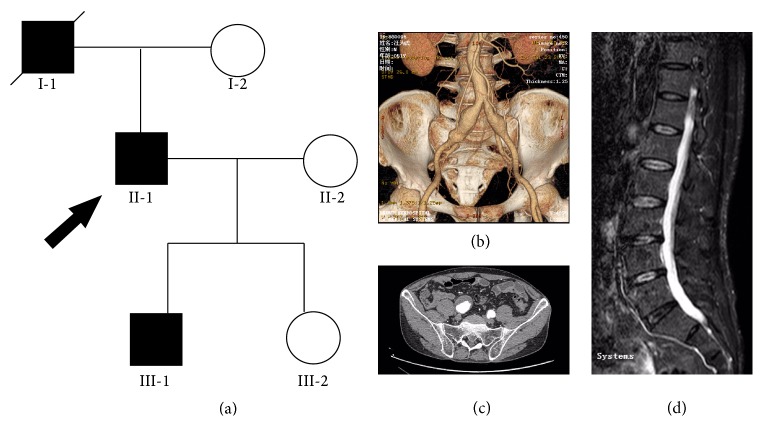
Pedigree and radiologic findings. (a) Pedigree of a Chinese family with aneurysms-osteoarthritis syndrome (AOS). Round symbols indicate female; square symbols, male; fully filled symbols, AOS; unfilled symbols, unaffected; diagonal lines, deceased; arrow, proband. (b) Computed tomography angiography (CTA) of II-2 demonstrated a bilateral common iliac artery aneurysm and abdominal aorta tortuosity. (c) CTA of II-2 revealed mural thrombosis in the right common iliac artery aneurysm. (d) Magnetic resonance image (MRI) of II-2 showed marked degenerative changes of lumbar spine and narrowing of the spinal cord.

The pedigree of a three-generation Chinese family with AOS is shown in Figure 1(a). The family exhibited an autosomal dominant inheritance pattern. Radiographic examination of the artery tree and joints was performed in family members. The study was approved by the local ethics committee. Informed written consent was obtained from all participants.

### 2.2. Mutation Detection

Genomic DNA was isolated from peripheral blood using the QIAamp DNA Blood Mini Kit (Qiagen) using standard protocols. All nine coding exons and exon-intron boundaries of* SMAD3* were amplified by polymerase chain reaction (PCR) with primers designed by the Primer3 program (Table 1). PCR products were purified and sequenced on the ABI PRISM 3730 automated sequencer (Applied Biosystems) using the BigDye terminator cycle sequencing method. Sites of variation were identified by comparison to the* SMAD3* gene GenBank reference sequence (NM_005902.3).

### 2.3. Bioinformatic Analysis

The multiple SMAD3 protein sequences across species were aligned using the program ClustalW2 (http://www.ebi.ac.uk/Tools/clustalw2/) [13]. The PolyPhen2 (Polymorphism Phenotyping, http://genetics.bwh.harvard.edu/pph2/) [14], SIFT (Sorting Intolerant From Tolerant, http://sift.jcvi.org/www/SIFT_enst_submit.html) [15], and MutationTaster (http://www.mutationtaster.org/) [16] programs were utilized to predict the effects of sequence variants on the function of the protein.

## 3. Results

The proband (II-1) is a 52-year-old male who was admitted to our department complaining of abdominal pulsatile mass. Computed tomography angiography (CTA) scan revealed a bilateral common iliac artery aneurysm and abdominal aorta tortuosity (Figure 1(b)). The right common iliac artery aneurysm was estimated to be 5.0 cm in diameter accompanied by mural thrombosis (Figure 1(c)). He also reported a ten-year history of back pain. He was subsequently referred to magnetic resonance image (MRI) evaluation, which showed marked degenerative changes of lumbar spine and narrowing of the spinal cord (Figure 1(d)). He underwent an endovascular repair of the aneurysm and the procedure was uneventful. Family history was positive for sudden death in the proband's father (I-1) at the age of 48, for which the underlying cause remained unknown. Subsequent screening of his children revealed prominent dilation of the ascending aorta in the 22-year-old son (III-1). Neither of his mother (I-2) and daughter (III-2) presented vascular anomalies at the time of evaluation.

**Figure 2:**
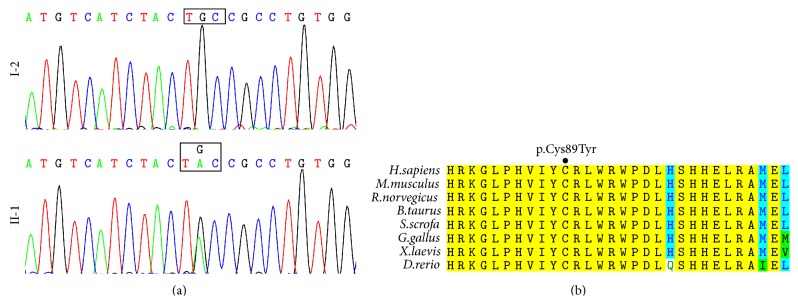
Mutation and bioinformatics analysis. (a) Sequencing results of the* SMAD3* mutation. Sequence chromatogram indicates a G to A transition of nucleotide 266, resulting in a transformation of Cysteine into Tyrosine at amino acid position 89. (b) Sequence alignment of SMAD3 protein shows highly conserved amino acid Cysteine across species.

*SMAD3* screening identified a heterozygous substitution of guanine to adenine at nucleotide 266 in the coding sequence of exon 2 (c.266G>A), in individuals II-1 and III-1 (Figure 2(a)). This variant resulted in a transformation of Cysteine into Tyrosine at amino acid position 89 (p.Cys89Tyr). It was not present in the unaffected individuals of the pedigree or in the 100 controls. Furthermore, this variant was not annotated in major databases, such as the Exome Sequencing Project, 1000 Genome, and dbSNP139. The altered amino acid is highly conserved across species (Figure 2(b)). Three programs for analyzing protein functions, PolyPhen2, SIFT, and MutationTaster, predicted that the p.C89Y variants are possibly damaging and disease causing, respectively. All three different algorithm based bioinformatics programs yield a consistent result of detrimental effect of the variant, suggesting that the site (C89) plays pivotal roles in the function of SMAD3.

## 4. Discussion

To the best of our knowledge, this is the first report of a Chinese family with AOS caused by a novel* SMAD3* mutation. AOS is a recently delineated autosomal dominant disorder characterized by aneurysms, dissections, and tortuosity throughout the arterial tree in association with early onset osteoarthritis, mild craniofacial features, and skeletal and cutaneous anomalies [2].* SMAD3* is the causative gene of AOS and may account for up to 2% of familial and nonfamilial thoracic aortic aneurysms and dissections (TAAD) [1]. Several lines of evidence support the notion that this variant is responsible for clinical phenotype of affected individuals in this family: (1) the sequence variant cosegregates with disease phenotype in the affected family members; (2) it is not reported in publicly available variation databases accessed in April 2015 and it is absent in ethnic-matched 200 control chromosomes; (3) the missense variant affects evolutionary highly conserved amino acid within the MH1 domain of SMAD3; (4) three computer programs which predict the possible impact of amino acid substitutions on the structure and function of human proteins (PolyPhen2, SIFT, and MutationTaster) indicate that the missense variant is pathogenic.

So far, 28 distinct exon mutations have been identified in the* SMAD3* gene, including the mutation identified in this study (Table 2). The mutations spread over the entire gene with marked increased rate in exon 6. The majority of them (19 of 29) are located in the MH2 domain, which are supposed to disrupt oligomerization of SMAD3 with SMAD4- and SMAD-dependent transcriptional activation. SMAD3 mutations lead to truncated protein or substitution of highly conserved amino acids, which are predicted* in silico* to have a deleterious effect. Besides exon mutations, splice-site mutations and large deletion of* SMAD3* gene have also been reported in AOS patients [11, 17]. With deeper understanding of the syndrome, novel mutations of* SMAD3* gene will be uncovered in near future. However, how to accurately distinguish deleterious variants from neutral ones is an issue that needs to be addressed. Although functional validation is the gold standard to confirm pathogenicity, the procedure is time consuming and laborious. Different types of computational algorithms have been developed to predict the effect of amino acid substitution [18]. Three basic prediction tools utilized in this study produced consistent and reliable results, suggesting that they are useful in identifying disease associated mutations. More advanced approaches such as molecular dynamics simulation have emerged as powerful methods with high accuracy and have already shown promising results in mutation analysis of cancer, neurodegenerative disorder, cardiomyopathy disease, and so on [19–21]. Further investigation of identified* SMAD3* mutations by these advanced tools is warranted to shed light on their pathogenic mechanisms.

It is noteworthy that significant overlap of clinical features is observed between AOS and other aorta aneurysm syndromes, especially LDS (caused by mutations in* TGFBR1* or* TGFBR2*) [5]. Similar to AOS, LDS is typically characterized by the triad of hypertelorism, cleft palate or bifid uvula, widespread arterial aneurysms, and tortuosity [22]. It is therefore challenging for clinicians to conduct specific gene screening according to aforementioned phenotype. In this study, we also performed mutation analysis of* TGFBR1* and* TGRBR2* in the proband. No pathologic mutation was identified in both genes. van de Laar et al. found the invariable presence of osteoarthritis at a young age in all patients with* SMAD3* mutations. Indeed, symptomatic osteoarthritis was the presenting complaint for them to seek medical advice [2]. In contrast, Regalado et al. reported a marked decreased incidence of osteoarthritis in AOS individuals. The diagnosis of osteoarthritis is based on interviews and medical records rather than systematic radiological evaluation, which is assumed to be responsible for the underestimate of incidence [1]. Aubart et al. reported skeleton involvement in 100% of* SMAD3* mutation carriers on X-ray or CT-scan study and recommended aorta screening in patients suffering from atypical osteoarthritis [4]. Recently, a patient with the* SMAD3 *mutation was demonstrated to have multiple aneurysms and rheumatoid arthritis [9]. In this study, the presence of both aneurysms and osteoarthritis prompted us to perform* SMAD3* screening in this family and find a causative mutation. Our results further strengthened the connection between this special phenotype and* SMAD3* mutation.

Given that mutation analysis of* SMAD3* was negative for the proband's mother, the variant, p.Cys89Tyr, was likely to be inherited from his father. His father was reported to be in good health until he suffered sudden death at the age of 48. The underlying cause could not be determined since postmortem examination was not performed. We highly speculated that his father carried this mutation and died of aneurysm rupture or dissection due to* SMAD3* mutation. Due to rapid aneurysmal growth and occurrence of dissections in only mildly dilated arteries, AOS is an aggressive disease with substantial mortality [5]. The estimated median survival in AOS is shorter than that in treated patients with MFS [23]. The proband developed aneurysm in the common iliac artery, which is a predilection site for aneurysm formation in AOS patients [24]. Given its aggressive behavior of AOS, early diagnosis and treatment are necessary. Endovascular repair seemed to be the first choice of treatment since it reduced the risk of major complication [24, 25]. Consistent with the observation, we performed stents implantation and achieved good results regarding the proband's treatment.

In conclusion, we identified a novel* SMAD3* sequence variant, segregating with the AOS phenotype in a Chinese family. This finding expands the mutation spectrum of* SMAD3* gene and highlights the importance of screening patients with aneurysms as well as early onset osteoarthritis for* SMAD3* mutation, which will facilitate identification of at-risk family members and early intervention.

## Figures and Tables

**Table 1:** Primers for PCR.

Exon	Forward (5′-3′)	Reverse (5′-3′)	Size
1a	AGAGTTGAGGCGAAGTTTGG	TGAGTTTCTTGACCAGGCTCTT	252
1b	GAGGAGAAATGGTGCGAGAAG	GATCTTTGCAAATCAGAGATGGTT	334
2	AAATGAGGGGAGAGAGAGCTT	ACCAACACAGGAGGTAGAACTG	488
3	ATCGACACTGAGCCACCTCT	AGTGTTGCTATTTCCGCTTCC	462
4	TGGTGTGCATGTGTGATGTC	ATCGCGGTTGCTCTACAAAT	299
5	CAGGGTTTTCTTTCTGCTGTG	GTTCTCAAGTTTCCCCATTCC	352
6	ACACCCAATGACCCAGTAGC	GAATGGAGCCACCCCATA	269
7	GCCATTGTGTGTGAGCAAAG	TGAGTGAGCAGAAAAGGTGAGA	292
8	CCAGGACTTGCTTTATCCAG	TCTTTGGTCTTTCTGCTCTTG	350
9	TGTCACCAAAGCAGAAAAAGC	CAATGGGTTGAGTAGAGTTCCA	302

**Table 2:** Exon mutations identified in the *SMAD3* gene.

ID	Mutation	Exon	Predicted protein change	Protein domain	Reference
1	c.3G>A	1	p.Met1Ile	MH1	[10]
2	c.266G>A	2	p.Cys89Tyr	MH1	This study
3	c.313delG	2	p.Ala105Profs^*∗*^11	MH1	[3]
4	c.335C>T	2	p.Ala112Val	MH1	[1]
5	c.401_405dup	3	p.Pro136Phefs^*∗*^52	MH1	[9]
6	c.539_540insC	4	p.Pro180Thrfs^*∗*^7	Linker	[3]
7	c.546delT	4	p.Gly183Alafs^*∗*^3	Linker	[11]
8	c.584_585insTC	4	p.Gln195Hisfs^*∗*^3	Linker	[11]
9	c.652delA	5	p.Asn218Thrfs^*∗*^23	Linker	[1]
10	c.668delC	6	p.Pro223Glnfs^*∗*^18	Linker	[4]
11	c.715G>A	6	p.Glu239Lys	MH2	[1, 8, 11]
12	c.733G>A	6	p.Gly245Arg	MH2	[4]
13	c.741_742delAT	6	p.Thr247Profs^*∗*^61	MH2	[2]
14	c.742T>C	6	p.Phe248Leu	MH2	[4]
15	c.782C>T	6	p.Thr261Ile	MH2	[2]
16	c.788C>T	6	p.Pro263Leu	MH2	[3]
17	c.836G>A	6	p.Arg279Lys	MH2	[1]
18	c.859C>T	6	p.Arg287Trp	MH2	[2, 11]
19	c.860G>A	6	p.Arg287Gln	MH2	[4]
20	c.862_871 + 1dup	6	p.Arg292Aspfs^*∗*^53	MH2	[4]
21	c.887T>C	7	p.Leu296Pro	MH2	[11]
22	c.1045G>C	8	p.Ala349Pro	MH2	[3]
23	c.1080dupT	8	p.Glu361^*∗*^	MH2	[3]
24	c.1102C>T	8	p.Arg368^*∗*^	MH2	[4]
25	c.1170_1179del	9	p.Ser391Alafs^*∗*^7	MH2	[12]
26	c.1179_1180dupC	9	p.Cys394Leufs^*∗*^4	MH2	[4]
27	c.1208C>T	9	p.Pro403Leu	MH2	[6]
28	c.1259G>A	9	p.Arg420His	MH2	[7]
29	c.1267A>G	9	p.Ser423Gly	MH2	[4]

The *SMAD3* reference sequence used was NM_005902.3, in which the A of the ATG translation initiation codon was nucleotide 1.
